# Highly Soluble Mussel Foot Protein and Its Derivatives Inhibit Inflammation by Targeting NF-κB/PI3K-Akt Signaling and Promoting M2 Macrophage Polarization

**DOI:** 10.3390/antiox14081021

**Published:** 2025-08-21

**Authors:** Na Li, Yu Li, Jiren Xu, Jeevithan Elango, Wenhui Wu

**Affiliations:** 1 Department of Marine Pharmacology, College of Food Science and Technology, Shanghai Ocean University, Shanghai 201306, China; d220300085@st.shou.edu.cn (N.L.); 2134228@st.shou.edu.cn (Y.L.); m240451270@st.shou.edu.cn (J.X.); 2 Department of Biomaterials Engineering, Faculty of Health Sciences, UCAM—Universidad Católica San Antonio de Murcia, Guadalupe, 30107 Murcia, Spain; 3 Marine Biomedical Science and Technology Innovation Platform of Lin-Gang Special Area, Shanghai 201306, China; 4 Putuo Branch of International Combined Research Center for Marine Biological Sciences, Zhoushan 316104, China

**Keywords:** chronic inflammation, mussel foot protein, anti-inflammatory activity, NF-κB signaling pathway, PI3K/Akt signaling pathway

## Abstract

Chronic inflammation is closely associated with various diseases, underscoring the need for natural, biocompatible anti-inflammatory candidates. For this purpose, mussel foot protein could be an excellent candidate due to its diverse biological activities. Hence, this study systematically evaluates the anti-inflammatory effects of a highly soluble mussel foot protein (HMFP) and HMFP-PEG using LPS-stimulated RAW264.7 cells as an in vitro inflammation model. The results reveal that both HMFP and HMFP-PEG markedly reduced intracellular reactive oxygen species (ROS) levels and suppressed the secretion of pro-inflammatory mediators, including IL-1β, TNF-α, and NO, while promoting the production of anti-inflammatory cytokines such as IL-10 and TGF-β. Mechanistically, both agents markedly inhibited the LPS-induced phosphorylation of PI3K, Akt, NF-κB, and IκB, indicating that their anti-inflammatory effects are mediated via suppression of the PI3K/Akt and NF-κB signaling pathways. Furthermore, HMFP and HMFP-PEG downregulated the expression of the inflammatory marker iNOS and markedly upregulated the M2 macrophage marker CD206, suggesting a role in promoting macrophage polarization toward an anti-inflammatory M2 phenotype. Notably, NF-κB signaling was identified as a key mediator in the anti-inflammatory mechanisms of both HMFP and its PEG-modified form. Collectively, these findings demonstrate that HMFP and HMFP-PEG exert significant anti-inflammatory effects through dual inhibition of NF-κB and PI3K/Akt signaling and by promoting M2 macrophage polarization, indicating their potential as promising candidates for the treatment of inflammation-related diseases.

## 1. Introduction

Inflammation is a critical defense response of the body to pathogen invasion, tissue damage, and stressful stimuli [1,2,3]. However, its prolonged activation often leads to chronic inflammation, which contributes to the development of various diseases, including cardiovascular diseases, neurodegenerative disorders, autoimmune diseases, and cancer [4,5,6]. Therefore, the development of anti-inflammatory candidates with good biosafety profiles and specific regulatory capabilities is of great significance in alleviating chronic inflammation-related diseases.

In recent years, naturally derived functional proteins have attracted widespread attention due to their excellent biocompatibility, biodegradability, and relatively low toxicity [7,8,9]. Mussel foot protein (MFP), a type of adhesive protein rich in dopamine-derived structures, exhibits unique structural characteristics that provide remarkable underwater adhesion, bioactivity, and functional expandability [10,11,12]. Among these, we previously obtained HMFP, which retains the structural advantages of MFP while offering improved water solubility and cellular compatibility, thus opening up new possibilities for biomedical applications [13]. However, there is currently a lack of systematic studies on whether HMFP possesses inherent anti-inflammatory properties. Furthermore, bifunctional polyethylene glycol (PEG) modification is widely used to enhance protein drug stability, prolong circulation time in vivo, and reduce immunogenicity [14,15,16].

The aim of this study is to elucidate the anti-inflammatory mechanisms of highly soluble mussel foot protein (HMFP) and its PEG-modified derivative (HMFP-PEG) using an LPS-stimulated RAW264.7 macrophage model. RAW264.7 is a murine macrophage-like cell line commonly used in immunological research, and lipopolysaccharide (LPS), a major component of Gram-negative bacterial cell walls, is widely employed to activate macrophages and simulate inflammatory responses in vitro. We systematically evaluated their regulatory potential by analyzing the expression of pro-inflammatory and anti-inflammatory cytokines, intracellular ROS levels, and the activation status of PI3K/Akt and NF-κB signaling pathways. This study was designed to determine whether HMFP and HMFP-PEG exert anti-inflammatory effects via modulation of these signaling pathways and macrophage polarization. This study provides mechanistic insights and theoretical support for the potential use of HMFP and HMFP-PEG as natural anti-inflammatory agents for treating inflammation-related diseases.

## 2. Materials and Methods

### 2.1. Materials and Reagents

HMFP and HMFP-PEG were prepared in our lab, with detailed extraction, purification, and PEGylation methods in published studies [13]. The CCK8 kit, ROS kit, IL-1β kit, TNF-α kit, IL-10 kit, TGF-β kit, and NO kit were purchased from Beyotime (Shanghai, China). PAGE gel preparation kits were purchased from Epizyme Biomedical Technology Co., Ltd. (Shanghai, China). Antibodies (PI3K, p-PI3K, Akt, p-Akt, NF-κB, p-NF-κB, IκB, p-IκB, iNOS, CD206, β-actin) were purchased from Huabio (Hangzhou, China). LPS (Lipopolysaccharide) was purchased from Sigma-Aldrich (Darmstadt, Germany). MG132 (Proteasome inhibitor) was purchased from Selleck Chemicals (Houston, TX, USA). Phosphate-buffered saline (PBS) washing buffer, Fetal bovine serum (FBS), Trypsin-EDTA solution, and Penicillin–Streptomycin solution (PS) (100×) were purchased from Gibco (Carlsbad, CA, USA). Dual-color protein standard marker (Catalog No. 1610374), 4× Laemmli Sample Buffer (Catalog No. 1610747), and 10× Tris/Glycine/SDS (Catalog No. 1610732) were purchased from Bio-Rad Laboratories Inc. (Hercules, CA, USA). Polyvinylidene fluoride (PVDF) membranes were purchased from Servicebio (Wuhan, China). RNA and qPCR-related reagents, including the SteadyPure Quick RNA Extraction Kit, Evo M-MLV RT Mix Kit with gDNA Clean for qPCR Ver.2, and SYBR Green Premix Pro Taq HS qPCR Kit, were purchased from Accurate Biotechnology Co., Ltd. (Changsha, China). Unless stated otherwise, all reagents were purchased from the Sigma-Aldrich Corporation (St. Louis, MO, USA).

### 2.2. Cell Viability Assay

Macrophage viability in response to HMFP and HMFP-PEG was evaluated using a CCK-8 assay. RAW264.7 cells were purchased from the Cell Bank of the Chinese Academy of Sciences (Shanghai, China) and cultured in Dulbecco’s Modified Eagle Medium (DMEM) supplemented with 10% fetal bovine serum (FBS) at 37 °C in a humidified atmosphere with 5% CO_2_. RAW264.7 cells were seeded in a 96-well plate at a density of 5 × 10^4^ cells/mL, with 100 µL per well, and cultured overnight at 37 °C in a 5% CO_2_ incubator. After replacing the culture medium containing different concentrations of HMFP or HMFP-PEG, the cells were incubated for an additional 24 h. Then, 10 µL of CCK-8 reagent was added to each well, and the cells were incubated for 2 h. Absorbance was measured at 450 nm using a microplate reader (BioTek, Winooski, VT, USA). Cell viability was calculated by comparing the optical density (OD) ratio of the treatment group to the blank control group.

### 2.3. ROS Measurement

To assess the effect of HMFP and HMFP-PEG on ROS levels in macrophages, the ROS fluorescent probe 2′,7′-dichlorodihydrofluorescein diacetate (DCFH-DA, Beyotime Biotechnology, Shanghai, China) was used for labeling, and intracellular ROS production was observed using a fluorescence microscope. RAW264.7 cells were seeded in a 24-well plate, and, after 24 h of treatment, the cells were washed with PBS. Then, 10 µM DCFH-DA working solution was added, and the cells were incubated at 37 °C for 30 min. After staining, the cells were washed again with PBS to remove the excess probe. Fluorescent images were captured using a fluorescence microscope (Olympus, Tokyo, Japan) under the FITC channel, and the green fluorescence intensity was employed to reflect the intracellular ROS levels.

### 2.4. Determination of Inflammatory Factors

The regulatory effect of HMFP and HMFP-PEG on macrophage inflammatory responses was evaluated using the ELISA method, measuring pro-inflammatory cytokines (IL-1β, TNF-α) and anti-inflammatory cytokines (IL-10, TGF-β). RAW264.7 cells were seeded in 24-well plates and treated under different conditions for 24 h. After incubation, the culture supernatant was collected and centrifuged to remove cell debris. ELISA kits were used to measure the cytokine levels according to the manufacturer’s instructions. The absorbance was read at 450 nm using a microplate reader (BIO-TEK, Inc., Winooski, VT, USA), and the cytokine concentrations were calculated.

### 2.5. Determination of NO Secretion Level

To assess the effect of HMFP and HMFP-PEG on NO secretion by macrophages, the Griess reagent assay was used. After treating RAW264.7 cells for 24 h, the culture supernatant was collected. A 50 μL aliquot of the supernatant was added to a 96-well plate, followed by an equal volume of Griess reagent, and incubated for 10 min. The absorbance (OD value) was measured at 540 nm using a microplate reader.

### 2.6. Quantitative Real-Time Polymerase Chain Reaction (qRT-PCR)

qRT-PCR is used for quantitatively analyzing the mRNA expression levels of genes [17]. Total RNA is first extracted from cells using TRIzol or similar reagents, ensuring that concentration and purity meet the standard (A260/A280 ratio of 1.8–2.0). The RNA is then converted into cDNA using a reverse transcription kit, with the reaction performed at 37 °C for 1 h, followed by enzyme inactivation at 95 °C. Then, a qPCR reaction is performed using SYBR Green or probe-labeled specific primers for amplification. Each reaction system contains an appropriate amount of cDNA, primers, SYBR Green, dNTPs, Taq enzyme, etc. The PCR reaction includes an initial denaturation (95 °C for 3 s), followed by 40 cycles of denaturation (95 °C for 15 s), annealing (55–60 °C for 20 s), and extension (72 °C for 30 s). Fluorescence signals are monitored in real-time, and the Ct values are calculated. The relative expression levels of the target gene are analyzed using the 2^−ΔΔCt^ method by comparing the Ct values of the target gene with those of the housekeeping gene. Finally, the results are used to analyze the changes in gene expression among the different groups. The specific primers were synthesized by Sangon Biotech, with sequences listed in Table 1.

### 2.7. Western Blot Analysis

Western blot analysis was used to detect protein expression levels and post-translational modifications [18]. First, total protein was extracted from RAW264.7 cells using RIPA lysis buffer, and protein concentration was quantified using the BCA method. Equal amounts of protein samples were mixed with loading buffer and heated before being loaded onto an SDS-PAGE gel for protein separation via electrophoresis. The separated proteins were then transferred onto a PVDF membrane and blocked with 5% skim milk at room temperature for 1 h. After transfer, the membrane was incubated overnight at 4 °C with primary antibodies specific to PI3K, p-PI3K, Akt, p-Akt, NF-κB, p-NF-κB, IκB, p-IκB, iNOS, and CD206. Following three washes with TBST, the membrane was incubated with secondary antibodies at room temperature for 1 h. After washing, the membrane was developed with an ECL chemiluminescent reagent, and chemiluminescent signals were captured and analyzed. Finally, the expression of target proteins was quantified using analysis software, such as ImageJ (version 1.54p), and normalized to internal reference proteins to evaluate the relative expression levels and changes in post-translational modifications, such as phosphorylation.

### 2.8. Statistical Analysis

Data are presented as the mean ± standard deviation (SD) from at least three independent experiments. Duncan’s multiple range test and one-way ANOVA were used to evaluate the significance of differences between group means, with the statistical analysis performed using SPSS 17.0 (SPSS, Inc., Chicago, IL, USA). Statistical significance compared with the model group is denoted by asterisks: * *p* < 0.05, ** *p* < 0.01, *** *p* < 0.001; Statistical significance between different treatment groups is denoted by hashes: # *p* < 0.05, ## *p* < 0.01, ### *p* < 0.001. Differences were considered statistically significant when *p* < 0.05.

## 3. Results

### 3.1. Effects of HMFP and HMFP-PEG on RAW264.7 Cell Viability and Intracellular ROS Levels

**Figure 1:**
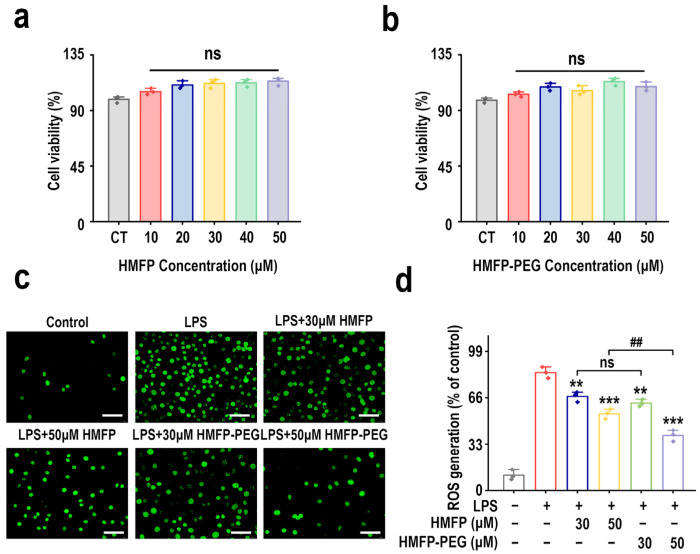
Effects of HMFP and HMFP-PEG on RAW264.7 cell viability and ROS generation. (**a**,**b**) CCK-8 assay assessing the effects of HMFP and HMFP-PEG (0–50 μM) on RAW264.7 cell viability. (**c**) Intracellular ROS levels detected using the DCFH-DA fluorescence probe (scale bar, 50 μm). (**d**) Quantitative analysis of fluorescence images from panel (**c**). Statistical significance: ** *p* < 0.01, *** *p* < 0.001 vs. model group; ^##^ *p* < 0.01 between groups; ns denotes no significant difference.

The effects of varying HMFP and HMFP-PEG concentrations (0, 10, 20, 30, 40, and 50 μM) on RAW264.7 macrophage viability were assessed using the CCK-8 assay. The results indicate that neither HMFP nor HMFP-PEG showed significant cytotoxicity within the tested concentration range (0–50 μM) (Figure 1a,b). Based on these findings, concentrations of 30 μM and 50 μM were selected for subsequent anti-inflammatory activity studies.

Reactive oxygen species (ROS) are key mediators in inflammatory responses, and anti-inflammatory activity is often achieved by inhibiting ROS production or facilitating ROS clearance [19,20,21]. To evaluate the anti-inflammatory potential of HMFP and HMFP-PEG, we used the DCFH-DA fluorescence probe to detect their effects on LPS-induced ROS generation in RAW264.7 cells. The experimental results show that LPS stimulation markedly elevated intracellular ROS levels, whereas pretreatment with both HMFP and HMFP-PEG effectively inhibited the excessive ROS release in a concentration-dependent manner (Figure 1c,d). Notably, at a concentration of 50 μM, HMFP-PEG revealed a stronger ROS suppression ability compared to HMFP, indicating that HMFP-PEG may exhibit superior anti-inflammatory effects.

### 3.2. HMFP and HMFP-PEG Inhibit Pro-Inflammatory Cytokine Expression and Promote Anti-Inflammatory Cytokine Secretion in RAW264.7 Cell

**Figure 2:**
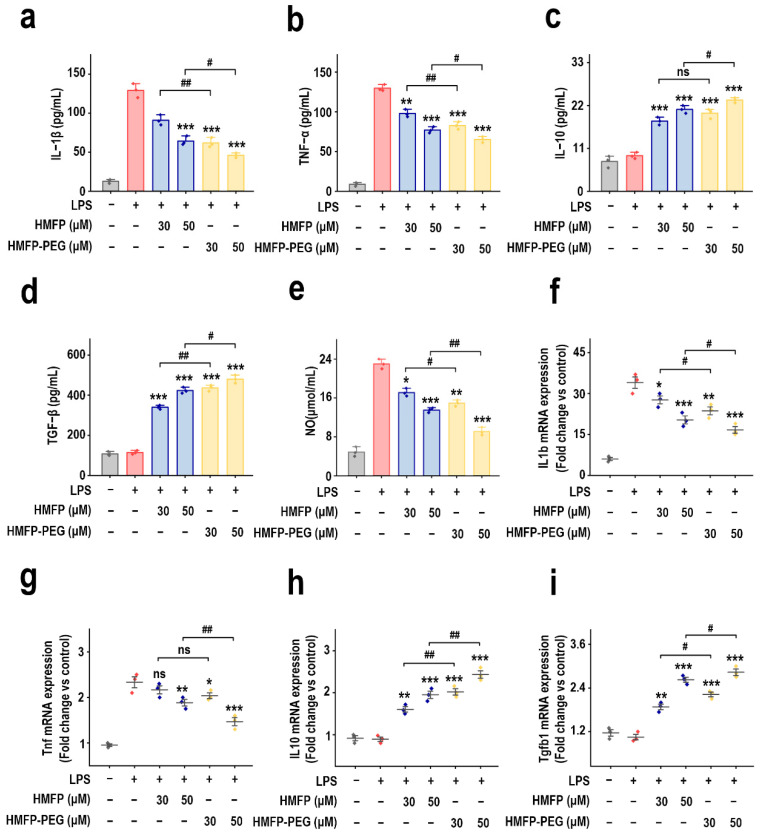
Effects of HMFP and HMFP-PEG on inflammatory cytokine secretion levels in RAW264.7 macrophages, including IL-1β (**a**), TNF-α (**b**), IL-10 (**c**), and TGF-β (**d**), as well as the NO production (**e**) and mRNA expression levels of IL1b (**f**), Tnf (**g**), IL10 (**h**), and Tgfb1 (**i**). *, **, and *** indicate *p* < 0.05, *p* < 0.01, and *p* < 0.001 compared to the model group, respectively. #, and ## represent significant differences between the two groups with *p* < 0.05, and *p* < 0.01, respectively; ns indicates no significant difference.

To further evaluate the anti-inflammatory activity of HMFP and its modified product HMFP-PEG, we measured the secretion levels and mRNA expression of typical pro-inflammatory factors (IL-1β, TNF-α, NO) and anti-inflammatory factors (IL-10, TGF-β) in RAW264.7 macrophages. The ELISA results show that LPS stimulation markedly induced the secretion of pro-inflammatory factors IL-1β and TNF-α (Figure 2a,b) and elevated NO release (Figure 2e). In contrast, both HMFP and HMFP-PEG treatments effectively inhibited the secretion of these pro-inflammatory factors in a concentration-dependent manner (Figure 2a,b,e). Notably, at a concentration of 50 μM, HMFP-PEG showed the most significant inhibitory effect.

Additionally, the secretion of anti-inflammatory factors IL-10 and TGF-β was suppressed by LPS, but treatment with HMFP and HMFP-PEG markedly elevated their secretion levels (Figure 2c,d). This trend was more pronounced in the high-concentration HMFP-PEG group, further validating its anti-inflammatory activity. The qPCR analysis results further supported these findings. LPS markedly upregulated the mRNA expression of pro-inflammatory factors IL1b and Tnf (Figure 2f,g), while both HMFP and HMFP-PEG markedly downregulated their expression. In comparison, HMFP-PEG showed a stronger inhibitory effect on the mRNA levels of IL1b and Tnf. Moreover, the mRNA expression of the anti-inflammatory factors IL10 and Tgfb1 was markedly elevated after treatment with HMFP and HMFP-PEG (Figure 2h,i). Overall, HMFP, especially HMFP-PEG, effectively alleviates LPS-induced inflammatory responses, likely through the inhibition of pro-inflammatory factors and the enhancement of anti-inflammatory factor expression.

### 3.3. HMFP and HMFP-PEG Inhibit Inflammation by Suppressing the PI3K-Akt Pathway

**Figure 3:**
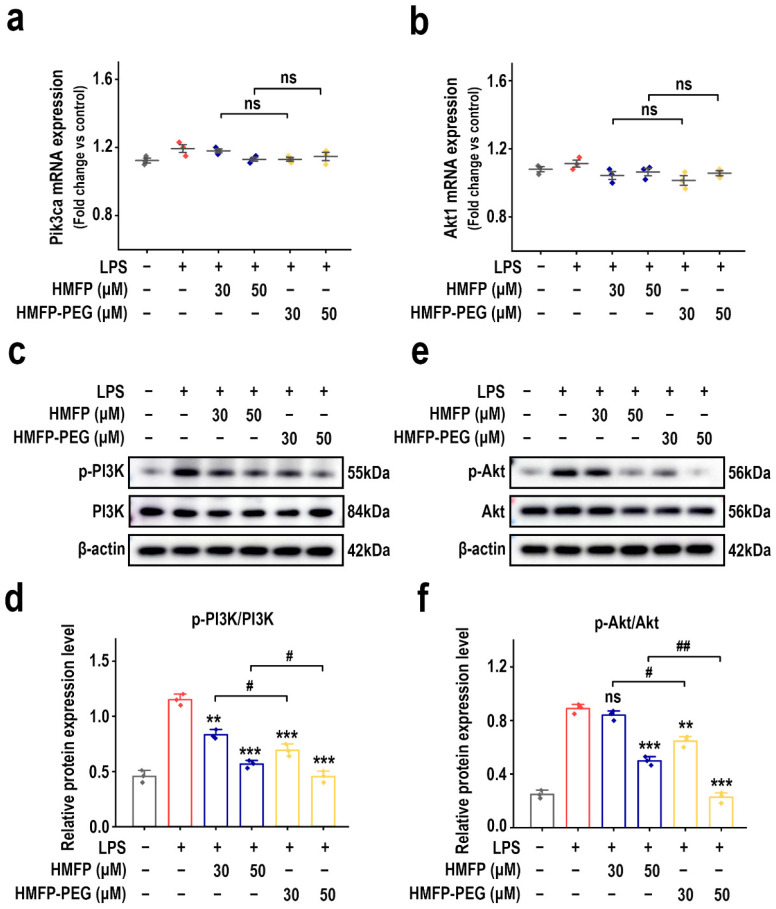
Effects of HMFP and HMFP-PEG on the PI3K-Akt signaling pathway in macrophages. (**a**,**b**) Analysis of mRNA expression levels of *Pik3ca* and *Akt1*. (**c**,**d**) Western blot analysis and quantification of PI3K and phosphorylated PI3K (p-PI3K) protein expression. (**e**,**f**) Western blot analysis and quantification of Akt and phosphorylated Akt (p-Akt) protein expression. ** and *** indicate *p* < 0.01, and *p* < 0.001 compared to the model group, respectively. # and ## represent significant differences between the two groups with *p* < 0.05 and *p* < 0.01, respectively; ns indicates no significant difference.

The PI3K-Akt signaling pathway plays a crucial role in regulating immune and inflammatory responses [22,23,24]. Its activation induces the production of pro-inflammatory factors and promotes macrophage polarization toward the M1 phenotype, thereby exacerbating the inflammatory response [25,26,27]. To further investigate whether the anti-inflammatory mechanism of HMFP and its derivative HMFP-PEG is related to the PI3K-Akt signaling pathway, we first examined the mRNA expression levels of *Pik3ca* and *Akt1*, the genes encoding PI3K and Akt, respectively. RT-qPCR results show that, after LPS induction, there was no significant difference in the mRNA levels of *Pik3ca* and *Akt1* compared to the control group, and no obvious changes were observed among the treatment groups, indicating stable transcriptional levels (Figure 3a,b).

Furthermore, protein phosphorylation levels were assessed using Western blot, and the results reveal that LPS markedly upregulated the expression of p-PI3K and p-Akt in RAW264.7 cells, thereby activating the pathway. However, HMFP and HMFP-PEG treatment effectively suppressed LPS-induced upregulation of p-PI3K and p-Akt (Figure 3c–f), with high-dose HMFP-PEG exhibiting the strongest inhibitory effect, suggesting the enhanced regulation of the PI3K-Akt pathway.

Overall, HMFP, particularly HMFP-PEG, likely exerts its anti-inflammatory effects by inhibiting the phosphorylation levels of PI3K and Akt, thereby suppressing the activation of the signaling pathway. This also provides a potential mechanistic basis for the negative regulation of the downstream NF-κB pathway.

### 3.4. HMFP and HMFP-PEG Alleviate Inflammation by Inhibiting the NF-κB Signaling Pathway

**Figure 4:**
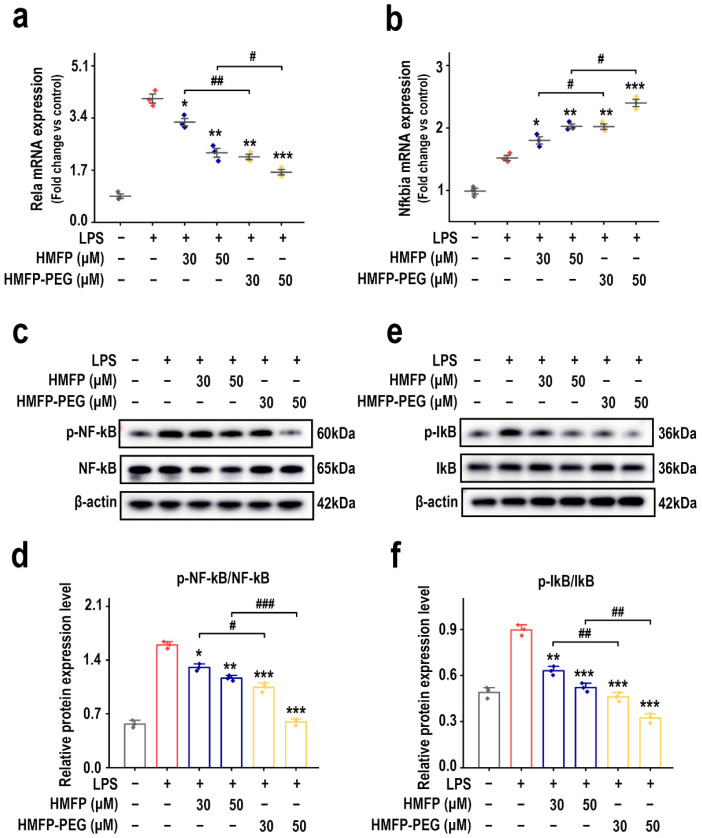
Effects of HMFP and HMFP-PEG on the NF-κB signaling pathway in macrophages. (**a**,**b**) Analysis of mRNA expression levels of *Rela* and *Nfkbia.* (**c**,**d**) Western blot analysis and quantification of NF-κB and phosphorylated NF-κB (p-NF-κB) protein expression. (**e**,**f**) Western blot analysis and quantification of IκB and phosphorylated IκB (p-IκB) protein expression. *, **, and *** indicate *p* < 0.05, *p* < 0.01, and *p* < 0.001 compared to the model group, respectively. #, ##, and ### represent significant differences between the two groups with *p* < 0.05, *p* < 0.01, and *p* < 0.001, respectively; ns indicates no significant difference.

Notably, the PI3K/Akt pathway, a key upstream regulator of NF-κB signaling, enhances NF-κB p65 phosphorylation and nuclear translocation, thereby driving the expression of pro-inflammatory factors [28,29,30]. Therefore, the regulatory effects of HMFP and HMFP-PEG on key proteins in the NF-κB signaling pathway were further evaluated. The results show that, in the model group, LPS treatment markedly elevated the mRNA levels of *Rela*, which encodes the p65 subunit of NF-κB, in macrophages (Figure 4a). However, following pre-treatment with HMFP and HMFP-PEG, the mRNA levels of *Rela* reduced markedly, indicating that HMFP and HMFP-PEG effectively inhibit NF-κB activation.

Although LPS treatment elevated the mRNA levels of Nfkbia, the gene encoding IκBα (Figure 4b), this increase was relatively modest compared to *Rela*, indicating that IκB, as a negative regulator of NF-κB, might be upregulated in response to feedback regulation of NF-κB activation. Further, Western blot analysis revealed that LPS markedly promoted the phosphorylation of NF-κB p65 and IκB, indicating activation of the NF-κB pathway. In contrast, HMFP and HMFP-PEG inhibited the expression of p-NF-κB p65 and p-IκB, indicating that they mitigate the inflammatory response by intervening in the NF-κB signaling pathway (Figure 4c–f).

Furthermore, the inhibitory effect of HMFP-PEG was more pronounced at higher concentrations, which may be attributed to the enhanced solubility and intracellular bioactivity provided by the PEG modification. By suppressing the activation of the NF-κB pathway, HMFP and HMFP-PEG alleviate LPS-induced inflammation, providing experimental evidence for their potential application in inflammation-related diseases.

### 3.5. HMFP and Its Derivatives Regulate iNOS/CD206 Expression and Promote RAW264.7 Cell Polarization Toward an Anti-Inflammatory Phenotype

**Figure 5:**
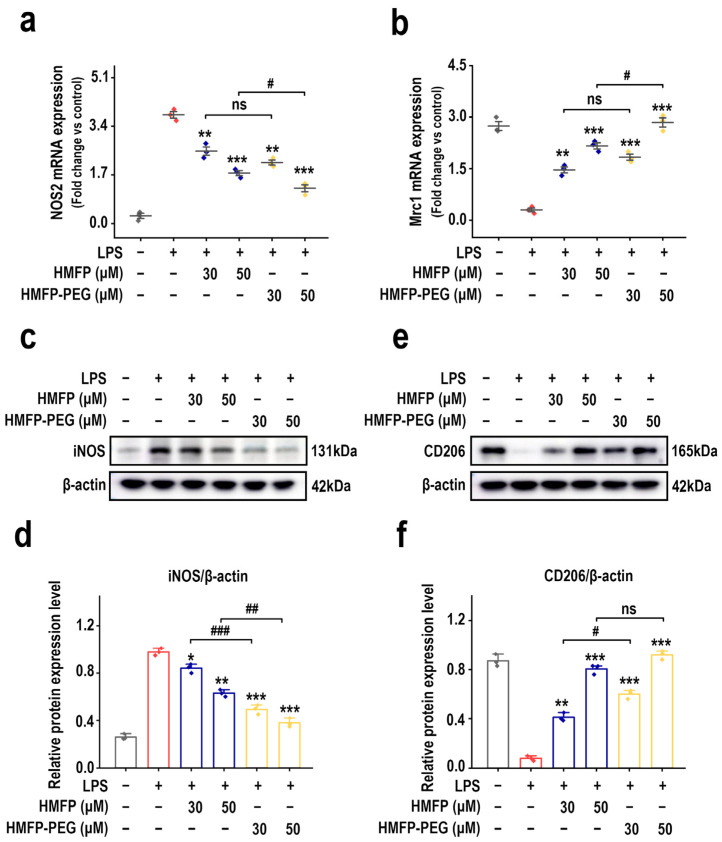
Regulatory effects of HMFP and HMFP-PEG on macrophage polarization. (**a**,**b**) Analysis of mRNA expression levels of NOS2 and Mrc1. (**c**,**d**) Western blot analysis and quantification of iNOS protein expression. (**e**,**f**) Western blot analysis and quantification of CD206 protein expression. *, **, and *** indicate *p* < 0.05, *p* < 0.01, and *p* < 0.001 compared to the model group, respectively. #, ##, and ### represent significant differences between the two groups with *p* < 0.05, *p* < 0.01, and *p* < 0.001, respectively; ns indicates no significant difference.

To further validate the regulatory effects of HMFP and HMFP-PEG on macrophage phenotype conversion, we examined the expression of M1 marker factor iNOS and M2 marker factor CD206. RT-qPCR results show that LPS stimulation markedly upregulated the expression of NOS2 (encoding iNOS), a marker gene for the pro-inflammatory M1 phenotype, while the expression of Mrc1 (encoding CD206), a marker gene for the anti-inflammatory M2 phenotype, was suppressed. In contrast, treatment with HMFP and HMFP-PEG showed the opposite trend, indicating that they may induce macrophage polarization toward the M2 phenotype (Figure 5a,b).

At the protein level, Western blot analysis further confirmed these findings. Compared to the LPS group, HMFP and HMFP-PEG markedly inhibited the expression of iNOS while upregulating the protein levels of CD206 (Figure 5c–f). Notably, HMFP-PEG showed a more pronounced regulatory effect at higher concentrations. These results indicate that HMFP and its derivatives have the potential to induce the conversion of macrophages from the pro-inflammatory M1 phenotype to the anti-inflammatory M2 phenotype, which may be one of the key mechanisms underlying their anti-inflammatory activity.

### 3.6. HMFP and HMFP-PEG Further Influence the Activity of the NF-κB Signaling Pathway Under the Effect of the NF-κB Pathway Inhibitor MG132

**Figure 6:**
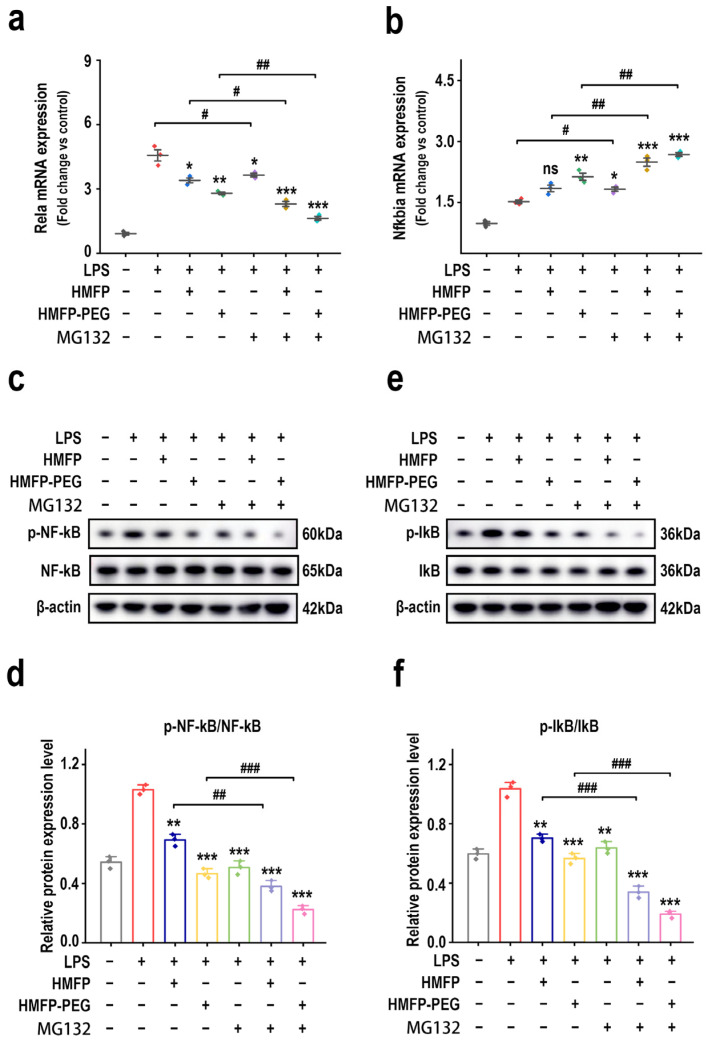
Effects of HMFP and HMFP-PEG on the NF-κB signaling pathway in macrophages after MG132 treatment. (**a**,**b**) Analysis of mRNA expression levels of *Rela* and *Nfkbia*. (**c**,**d**) Western blot analysis and quantification of NF-κB and p-NF-κB protein expression. (**e**,**f**) Western blot analysis and quantification of IκB and p-IκB protein expression. *, **, and *** indicate *p* < 0.05, *p* < 0.01, and *p* < 0.001 compared to the model group, respectively. #, ##, and ### represent significant differences between the two groups with *p* < 0.05, *p* < 0.01, and *p* < 0.001, respectively; ns indicates no significant difference.

To further verify whether HMFP and HMFP-PEG exert their anti-inflammatory effects through the NF-κB pathway, we combined the use of the NF-κB pathway inhibitor MG132 and assessed the expression levels of key pathway components [31]. RT-qPCR results indicate that LPS stimulation markedly induced the transcriptional expression of NF-κB (Rela) and its downstream inhibitory protein IκBα (Nfkbia). After treatment with MG132, the mRNA expression of Rela was markedly downregulated, while Nfkbia expression was upregulated (Figure 6a,b). These results suggest that MG132 differentially regulates the pathway components at the transcriptional level: Rela is suppressed, while IκBα is further induced, possibly through a negative feedback mechanism.

Western blot analysis further confirmed that, at the protein level, MG132 treatment markedly inhibited the phosphorylation of NF-κB p65 and IκBα (p-NF-κB, p-IκB), indicating the effective suppression of the pathway activation. Treatment with HMFP and HMFP-PEG in combination with MG132 further enhanced this inhibitory effect (Figure 6c–f), with the most pronounced suppression of phosphorylation observed in the high-dose HMFP-PEG group.

Taken together, these results suggest that HMFP and HMFP-PEG can intervene in the NF-κB signaling pathway through multi-level regulatory mechanisms (including transcription and protein phosphorylation), and that the combined action with MG132 further inhibits inflammatory signal transduction, thereby promoting their anti-inflammatory activity.

### 3.7. Analysis of Changes in the PI3K/Akt Signaling Pathway Mediated by the NF-κB Signaling Pathway Inhibitor

**Figure 7:**
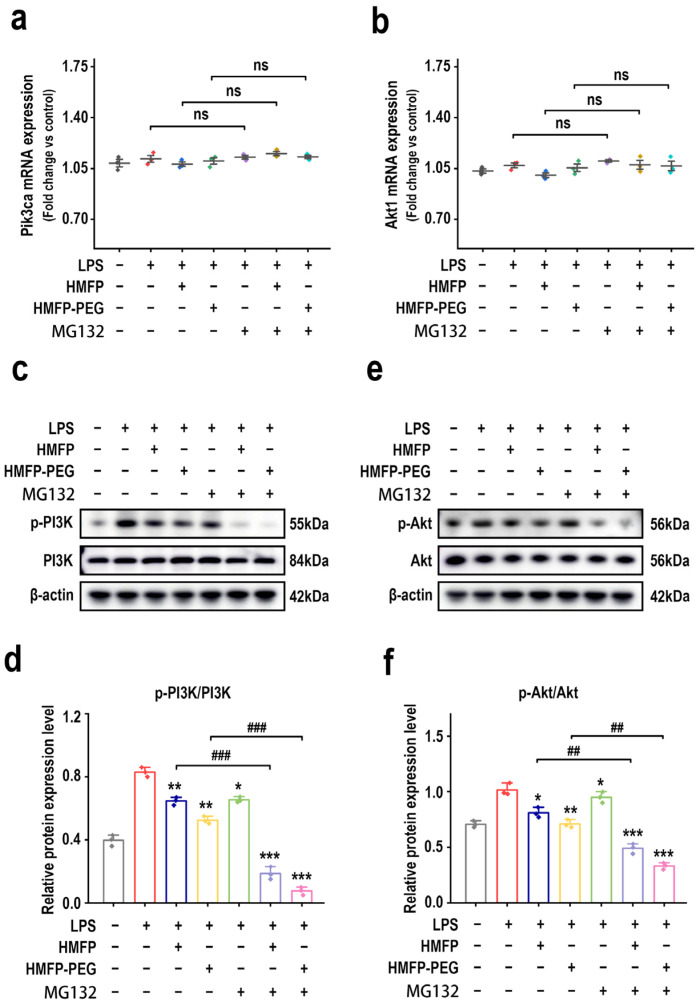
Effects of HMFP and HMFP-PEG on the PI3K-Akt signaling pathway in macrophages after MG132 treatment. (**a**,**b**) Analysis of mRNA expression levels of *Pik3ca* and *Akt1*. (**c**,**d**) Western blot analysis and quantification of PI3K and p-PI3K protein expression. (**e**,**f**) Western blot analysis and quantification of Akt and p-Akt protein expression. *, **, and *** indicate *p* < 0.05, *p* < 0.01, and *p* < 0.001 compared to the model group, respectively. ^##^ and ^###^ represent significant differences between the two groups with *p* < 0.01, and *p* < 0.001, respectively; ns indicates no significant difference.

To further investigate the role of the NF-κB pathway in the inhibition of PI3K/Akt signaling by HMFP and HMFP-PEG, the NF-κB inhibitor MG132 was added to the cells, and the expression levels of PI3K and Akt were assessed. RT-qPCR results show that, after MG132 treatment, there were no significant differences in the mRNA levels of *Pik3ca* and *Akt1* across the groups (Figure 7a,b), indicating that this process primarily affects post-translational modifications rather than gene transcription levels.

Subsequently, the phosphorylation levels of PI3K and Akt were measured using Western blot. The results show that, compared to the LPS-only group, MG132 treatment markedly reduced the expression of p-PI3K and p-Akt (Figure 7c–f), indicating that inhibition of the NF-κB signaling pathway suppresses the activation of the PI3K/Akt pathway.

Further analysis showed that MG132 treatment enhanced the downregulation of p-PI3K and p-Akt in HMFP and HMFP-PEG groups, with the most pronounced inhibition observed in the high-dose HMFP-PEG group. These results suggest that HMFP and HMFP-PEG may partially exert their anti-inflammatory effects by inhibiting the NF-κB pathway, thereby affecting the activity of the PI3K/Akt signaling pathway.

### 3.8. Regulation of Macrophage Polarization Markers by HMFP and HMFP-PEG Under MG132 Intervention

**Figure 8:**
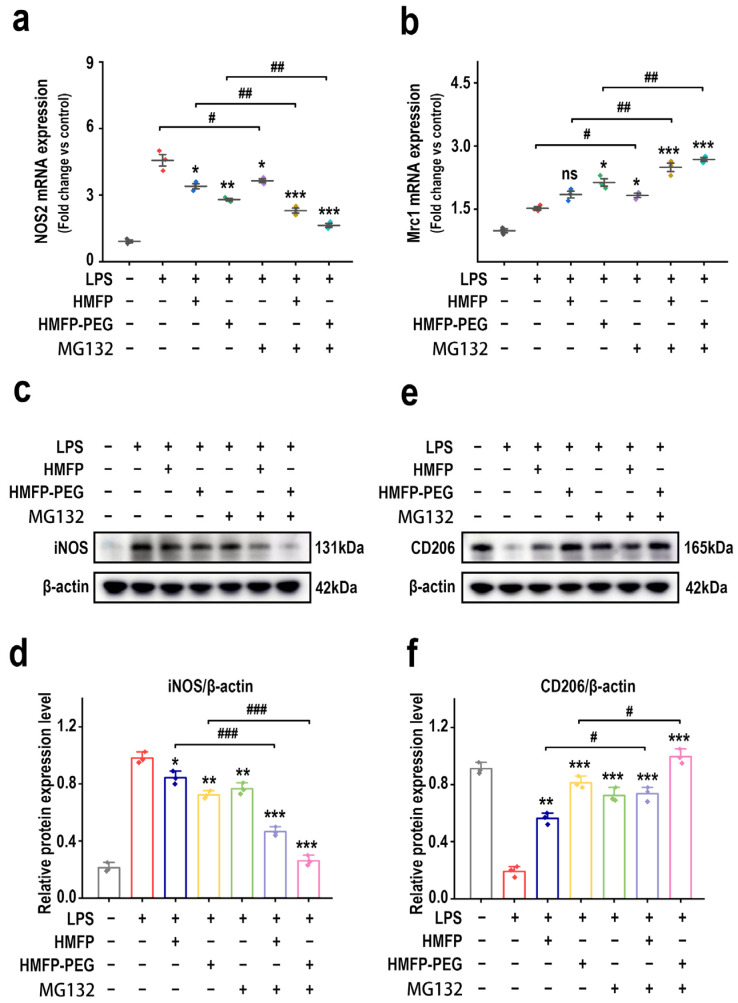
Effects of HMFP and HMFP-PEG on macrophage polarization after MG132 treatment. (**a**,**b**) Analysis of the mRNA expression levels of *NOS2* and *Mrc1*. (**c**,**d**) Western blot analysis and quantification of iNOS protein expression. (**e**,**f**) Western blot analysis and quantification of CD206 protein expression. *, **, and *** indicate *p* < 0.05, *p* < 0.01, and *p* < 0.001 compared to the model group, respectively. #, ##, and ### represent significant differences between the two groups with *p* < 0.05, *p* < 0.01, and *p* < 0.001, respectively; ns indicates no significant difference.

To explore the link between the anti-inflammatory activity of HMFP and HMFP-PEG and macrophage polarization, the expression of the pro-inflammatory (M1) marker gene iNOS (NOS2) and the anti-inflammatory (M2) marker gene CD206 (Mrc1) under NF-κB pathway inhibition was analyzed. RT-qPCR results show that co-treatment with MG132 and HMFP or HMFP-PEG markedly downregulated NOS2 mRNA levels while markedly upregulating Mrc1 expression, suggesting the suppression of the pro-inflammatory phenotype and the enhancement of the anti-inflammatory phenotype (Figure 8a,b).

At the protein level, Western blot results further confirm this trend. MG132 treatment enhanced the inhibitory effect of HMFP and HMFP-PEG on iNOS expression and further elevated CD206 protein levels (Figure 8c–f). These findings indicate that inhibition of the NF-κB pathway not only helps suppress the inflammatory response, but also synergistically enhances the ability of HMFP and HMFP-PEG to promote macrophage polarization toward the M2 phenotype, thereby contributing to their anti-inflammatory effects through multi-pathway regulation.

## 4. Discussion

In recent years, natural proteins have attracted increasing attention in anti-inflammatory drug development due to their excellent biocompatibility and functional diversity [32,33,34]. Among them, mussel foot protein (MFP), derived from marine organisms, has been widely applied in tissue engineering, hemostatic materials, and surface coatings, owing to its unique catechol structure and strong adhesive properties [35,36,37]. However, whether MFP possesses anti-inflammatory activity—particularly its potential to modulate inflammatory responses at the cellular level—remains largely unexplored. In this study, we systematically evaluate the anti-inflammatory potential of highly soluble mussel foot protein (HMFP) and its PEGylated derivative (HMFP-PEG).

Our results show that both HMFP and HMFP-PEG showed no cytotoxicity toward RAW264.7 macrophages at concentrations ranging from 1 to 50 μM. Notably, pre-treatment with HMFP or HMFP-PEG markedly reduced intracellular reactive oxygen species (ROS) levels upon lipopolysaccharide (LPS) stimulation compared to the model group (Figure 1). As ROS act as upstream mediators in pro-inflammatory signaling, triggering pathways such as MAPK and NF-κB and promoting excessive release of pro-inflammatory cytokines, the observed ROS suppression suggests that HMFP may exert a preliminary radical scavenging effect or indirect antioxidant activity.

Inflammation is a defensive response of the body to infection or tissue injury [38,39,40]. Pro-inflammatory mediators, such as IL-1β, TNF-α, and nitric oxide (NO), produced via inducible nitric oxide synthase (iNOS), are rapidly released during early inflammation, activating macrophages, stimulating endothelial cells, and recruiting additional immune cells to amplify the inflammatory cascade [41,42,43]. In contrast, anti-inflammatory cytokines such as IL-10 and TGF-β serve to suppress the release of pro-inflammatory mediators and ROS, thus resolving inflammation and promoting tissue repair [44,45,46]. Maintaining a balanced cytokine profile is critical for immune homeostasis, and the associated signaling pathways are key targets for anti-inflammatory intervention [47,48,49]. In LPS-stimulated RAW264.7 cells, HMFP and HMFP-PEG markedly reduced the secretion of IL-1β, TNF-α, and NO, while upregulating IL-10 and TGF-β expression at both mRNA and protein levels (Figure 2). This dual regulatory effect contributes to the attenuation of pro-inflammatory amplification and the restoration of inflammatory microenvironmental balance.

To further elucidate the underlying mechanisms, we focused on two critical inflammatory signaling pathways, PI3K/Akt and NF-κB, both of which play pivotal roles in macrophage polarization [26,50]. The PI3K/Akt pathway is known to act as an upstream activator of NF-κB in various inflammation models [30]. Upon LPS stimulation, phosphorylated PI3K (p-PI3K) and Akt (p-Akt) levels were markedly elevated, whereas treatment with HMFP or HMFP-PEG markedly suppressed this activation (Figure 3), indicating their potential to interfere with early inflammatory signaling events.

NF-κB, a central transcription factor in inflammation, translocates into the nucleus upon activation by upstream signals such as PI3K/Akt and induces the transcription of pro-inflammatory genes including IL-1β, TNF-α, and iNOS [51,52,53]. Our results show that HMFP and HMFP-PEG effectively inhibited the phosphorylation of NF-κB p65 and IκBα (Figure 4), indicating that the anti-inflammatory effect of HMFP is mediated, at least in part, via the NF-κB pathway. To validate the functional role of NF-κB in mediating the observed anti-inflammatory responses, we assessed the expression of iNOS and CD206, markers of M1 (pro-inflammatory) and M2 (anti-inflammatory) macrophage phenotypes, respectively [54]. HMFP and HMFP-PEG markedly downregulated iNOS expression while upregulating CD206 levels (Figure 5), demonstrating their capacity to promote M2 polarization and shift macrophages toward an anti-inflammatory state, consistent with NF-κB pathway inhibition.

To further substantiate the central role of NF-κB in HMFP-mediated anti-inflammatory activity, we employed the NF-κB inhibitor MG132 to evaluate its impact on relevant signaling and phenotypic markers [31]. MG132 effectively prevented IκB degradation and suppressed the LPS-induced phosphorylation of NF-κB p65 and IκBα (Figure 6), confirming the successful blockade of the NF-κB axis. Importantly, in the presence of MG132, the inhibitory effects of HMFP and HMFP-PEG on the NF-κB pathway were further enhanced, reinforcing the conclusion that their anti-inflammatory activity is largely dependent on NF-κB suppression.

Moreover, the phosphorylation levels of PI3K and Akt were also reduced upon MG132 treatment, indicating that the PI3K/Akt pathway may act in coordination with or downstream of NF-κB in regulating inflammatory responses (Figure 7). These findings support a model in which HMFP and HMFP-PEG exert anti-inflammatory effects through multi-level regulation of interconnected signaling pathways.

At the effector level, the combination of MG132 with HMFP or HMFP-PEG led to more pronounced suppression of iNOS expression and enhancement of CD206 expression (Figure 8), confirming that inhibition of the NF-κB pathway not only blocks upstream inflammatory signaling, but also promotes phenotypic reprogramming toward an anti-inflammatory macrophage profile. These effects were consistent across both mRNA and protein levels, further underscoring the centrality of NF-κB and its crosstalk with PI3K/Akt in the observed bioactivity.

Notably, HMFP-PEG revealed superior anti-inflammatory efficacy compared to unmodified HMFP in several aspects, including ROS scavenging, signaling inhibition, and CD206 upregulation. PEGylation is well-known to improve protein stability, solubility, and cellular uptake, while extending intracellular retention time, thereby promoting bioactivity [55,56,57]. Thus, the enhanced performance of HMFP-PEG aligns with the expected structure–function relationship and highlights PEGylation as a promising strategy in the development of protein-based therapeutics. These properties suggest strong potential for translation into topical or systemic treatments for chronic inflammatory conditions, such as inflammatory bowel disease, arthritis, or wound healing applications.

## 5. Conclusions

**Figure 9:**
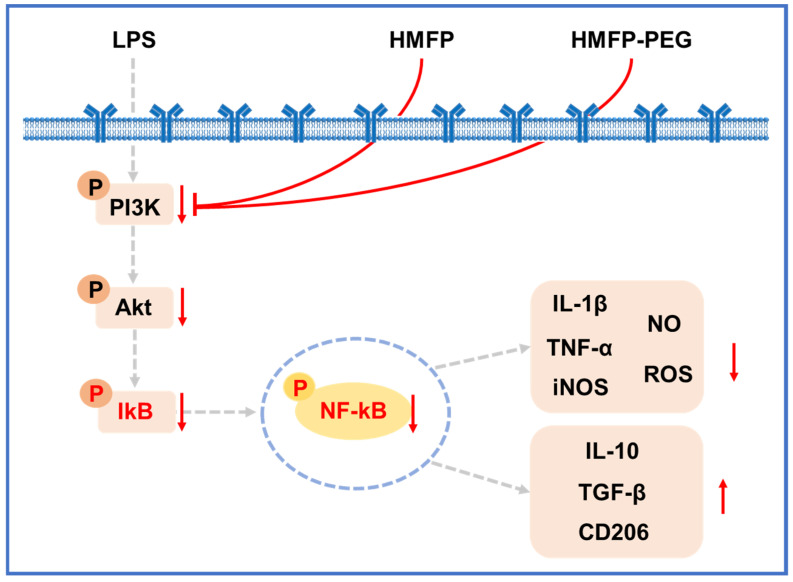
Proposed mechanism of the anti-inflammatory effects of HMFP and HMFP-PEG via inhibition of the PI3K/Akt/NF-κB pathway and promotion of M2 macrophage polarization.

This study comprehensively investigates the anti-inflammatory effects and underlying mechanisms of HMFP and its PEG-modified derivative (HMFP-PEG). Using LPS-stimulated RAW264.7 macrophages as an in vitro inflammation model, we reveal that both HMFP and HMFP-PEG effectively suppress the accumulation of reactive oxygen species (ROS), reduce the expression of pro-inflammatory mediators such as IL-1β, TNF-α, and nitric oxide (NO), and simultaneously promote the secretion of anti-inflammatory cytokines, including IL-10 and TGF-β, indicating their potent anti-inflammatory potential. Mechanistic investigations revealed that these effects were mediated through the inhibition of NF-κB and PI3K/Akt signaling pathway activation, the suppression of iNOS expression, and the upregulation of CD206, thereby promoting macrophage polarization from the pro-inflammatory M1 phenotype toward the anti-inflammatory M2 phenotype (Figure 9). Furthermore, co-treatment with the NF-κB inhibitor MG132 amplified the anti-inflammatory activities of HMFP and HMFP-PEG, confirming the central role of the NF-κB signaling axis in their mechanism of action. Collectively, these findings highlight the promising bioactivity and chemical modifiability of HMFP and HMFP-PEG, supporting their potential as novel, natural anti-inflammatory candidates for further development. Given their biocompatibility, modifiability, and potent multi-pathway activity, HMFP and HMFP-PEG hold promise for future clinical applications in the treatment of chronic inflammation-associated diseases.

## Figures and Tables

**Table 1:** Primer sequences in RT-PCR used in the measurement of mRNA expression.

Gene	Accession Number	Primer Sequence
Il1b	NM_008361.4	Forward: 5′-TGGACCTTCCAGGATGAGGACA-3′
		Reverse: 5′-GTTCATCTCGGAGCCTGTAGTG-3′
Tnf	NM_013693.4	Forward: 5′-GGTGCCTATGTCTCAGCCTCTT-3′
		Reverse: 5′-GCCATAGAACTGATGAGAGGGAG-3′
Il10	NM_010548.5	Forward: 5′-CGGGAAGACAATAACTGCACCC-3′
		Reverse: 5′-CGGTTAGCAGTATGTTGTCCAGC-3′
Tgfb1	NM_011577.3	Forward: 5′-CCATGGATGCCGCCCTCGGG-3′
		Reverse: 5′-GCGGAAGTCAATGTACAGCTGCC-3′
Pik3ca	NM_008839.3	Forward: 5′-GAAGCACCTGAATAGGCAAGTCG-3′
		Reverse: 5′-GAGCATCCATGAAATCTGGTCGC-3′
Akt1	NM_009652.6	Forward: 5′-GGACTACTTGCACTCCGAGAAG-3′
		Reverse: 5′-CATAGTGGCACCGTCCTTGATC-3′
Rela	NM_009045.3	Forward: 5′-TCCTGTTCGAGTCTCCATGCAG-3′
		Reverse: 5′-GGTCTCATAGGTCCTTTTGCGC-3′
Nfkbia	NM_010907.2	Forward: 5′-GCTGCCAAAGAAGGACACGACA-3′
		Reverse: 5′-GGCAGGCTATTGCTCATCACAG-3′
NOS2	NM_010927.3	Forward: 5′-GAGACAGGGAAGTCTGAAGCAC-3′
		Reverse: 5′-CCAGCAGTAGTTGCTCCTCTTC-3′
Mrc1	NM_008625.2	Forward: 5′-GTTCACCTGGAGTGATGGTTCTC-3′
		Reverse: 5′- AGGACATGCCAGGGTCACCTTT-3′
β-Actin	NM_007393.5	Forward: 5′-GCTACAGCTTCACCACCACA-3′
		Reverse: 5′-AAGGAAGGCTGGAAAAGAGC-3′

## Data Availability

Data will be made available on request.
